# Population‐wide copy number variation calling using variant call format files from 6,898 individuals

**DOI:** 10.1002/gepi.22260

**Published:** 2019-09-14

**Authors:** Grace Png, Daniel Suveges, Young‐Chan Park, Klaudia Walter, Kousik Kundu, Ioanna Ntalla, Emmanouil Tsafantakis, Maria Karaleftheri, George Dedoussis, Eleftheria Zeggini, Arthur Gilly

**Affiliations:** ^1^ Wellcome Sanger Institute Wellcome Genome Campus Hinxton United Kingdom; ^2^ Department of Medical Genetics University of Cambridge Cambridge United Kingdom; ^3^ Institute of Translational Genomics Helmholtz Zentrum München—German Research Center for Environmental Health Neuherberg Germany; ^4^ European Bioinformatics Institute Wellcome Genome Campus Hinxton United Kingdom; ^5^ William Harvey Research Institute, Barts and The London School of Medicine and Dentistry Queen Mary University of London London United Kingdom; ^6^ Anogia Medical Centre Anogia Greece; ^7^ Echinos Medical Centre Echinos Greece; ^8^ Department of Nutrition and Dietetics, School of Health Science and Education Harokopio University of Athens Athens Greece; ^9^ Department of Public Health and Primary Care University of Cambridge Cambridge United Kingdom

**Keywords:** association study, copy‐number variant, whole‐genome sequencing

## Abstract

Copy number variants (CNVs) play an important role in a number of human diseases, but the accurate calling of CNVs remains challenging. Most current approaches to CNV detection use raw read alignments, which are computationally intensive to process. We use a regression tree‐based approach to call germline CNVs from whole‐genome sequencing (WGS, >18x) variant call sets in 6,898 samples across four European cohorts, and describe a rich large variation landscape comprising 1,320 CNVs. Eighty‐one percent of detected events have been previously reported in the Database of Genomic Variants. Twenty‐three percent of high‐quality deletions affect entire genes, and we recapitulate known events such as the *GSTM1* and *RHD* gene deletions. We test for association between the detected deletions and 275 protein levels in 1,457 individuals to assess the potential clinical impact of the detected CNVs. We describe complex CNV patterns underlying an association with levels of the CCL3 protein (MAF = 0.15, *p* = 3.6x10^−12^) at the *CCL3L3* locus, and a novel *cis‐*association between a low‐frequency *NOMO1* deletion and NOMO1 protein levels (MAF = 0.02, *p* = 2.2x10^−7^). This study demonstrates that existing population‐wide WGS call sets can be mined for germline CNVs with minimal computational overhead, delivering insight into a less well‐studied, yet potentially impactful class of genetic variant.

## 1 INTRODUCTION

Up to 19.2% of the human genome is susceptible to copy number variation, which can have a severe impact on gene function (Zarrei, MacDonald, Merico, & Scherer, 2015). Copy number variant (CNV) calling can be performed for individuals or families in a clinical context, or for large sample sizes in population cohorts. Whole‐genome sequencing (WGS) at high depth has been the gold standard for detecting large polymorphisms in population studies and is starting to replace array‐based calling in the clinic. Yet, calling structural variants genome‐wide has been an ongoing challenge throughout the history of computational genetics, and producing population‐wide CNV call sets still represents a significant investment today. The reasons for this are two‐fold. First, detecting structural variants requires a different study design compared with association studies: whereas for the latter, haplotype diversity and hence sample size are key (Alex Buerkle & Gompert, 2013; Le & Durbin, 2011), for the former, high depth of sequencing is paramount, leading to prohibitive costs for population‐wide studies. This is in addition to other upstream processing features, such as insert size, polymerase chain reaction (PCR) amplification bias, and choice of mapping software and reference genome, that also influence structural variant detection sensitivity (Trost et al., 2018). Second, structural variant detection poses a computational challenge, since most algorithms use aligned reads or read pileups as a starting point for event detection. As these file formats describe the entire read pool, processing them genome‐wide across an entire population with high‐depth WGS is demanding in terms of both running time and memory. CNV calling pipelines involving a combination of read depth and insert‐size based tools are increasingly included in analysis pipelines for large human population cohorts, however, the computing requirements and complexity of such methods often preclude their use in other settings. This is especially true when CNV calling algorithms were not integrated in standard WGS processing pipelines from the get‐go, in which case the entire read pool needs to be reprocessed again to produce a CNV callset. This issue can be addressed by detecting deletions and insertions from existing variant call sets, which demands much less compute effort. Such methods were pioneered in the era of genotyping chips (PennCNV; Wang et al., 2007) and PlatinumCNV (Kumasaka et al., 2011), are still widely used (Kayser et al., 2018; Selvanayagam et al., 2018) and have recently been proposed to call CNVs from marker‐level data in paired cancer samples (Putnam et al., 2017). To our knowledge, no such method exists for variant calls produced from population‐scale WGS (do Nascimento & Guimaraes, 2017). Such variant call sets are typically produced in the variant call format (VCF) in most association‐focused studies, and analysis of these comparably small files for CNV calling would be computationally efficient. Here, we evaluate the effect of CNVs on sequencing depth measured at variant sites using a novel tool (Unimaginatively Named CNV caller [UN‐CNVc]), and provide a proof‐of‐concept for calling these large variations in population‐wide WGS variant call sets.

## 2 MATERIALS AND METHODS

The observed read depth for a single sample in a WGS experiment can be modelled as a noisy piecewise constant function:

$$\hat{d}(x)=\sum_{k}k.1_{d(x)=k}(x)+\epsilon$$

where d(x)=0.5n is the ideal relative depth at position x, n is the copy number at this position, $1_{d(x)=k}\left(x\right)=\left\{ \begin{array}{cc} 1 & \mathrm{if d}(x)=k\\ 0 & \text{otherwise} \end{array}\right.$ is the indicator function for copy number k genome‐wide and ϵ ~ N(0,σ) is the error in estimating true read counts. This error term captures all non‐CNV factors influencing read depth, such as GC content or reference sequence quality. These variations tend to act on a short‐range, and over long stretches of sequence, average depths vary little around the per‐sample mean (Figure S1).

**Figure 1:**
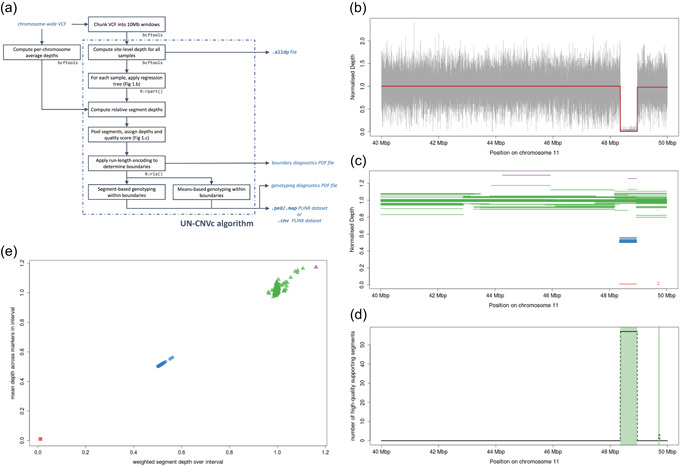
Overview of the UN‐CNVc algorithm. (a) Overview of the pipeline, with input and output files in blue, and external tools and libraries in grey. (b) Output of a piecewise constant regression (in red) on a 10Mb window on chromosome 11, for a homozygous deletion carrier. The gray signal is the raw relative depth at every sequenced marker for that sample. (c) Pooled regressed segments across the population, with colour indicating the attributed ideal depth (0: red, 0.5: blue, 1: green, and 1.5: purple). (d) Raw count (dashed line) and run‐length encoding (shaded green bars) on the number of high‐quality segments with ideal depth <1. (e) Genotyping using both weighted average segment depth (colour, scheme identical to c) and average depth across markers (plotting glyphs, squares: 0, circles: 0.5, and triangles: 1). UN‐CNVc, Unimaginatively Named CNV caller

Methods for fitting piecewise constant functions for CNV detection have included circular binary segmentation (Olshen, Venkatraman, Lucito, & Wigler, 2004; Venkatraman & Olshen, 2007), hidden Markov models (Seiser & Innocenti, 2014), smoothing approaches (Hsu, 2005; Tibshirani & Wang, 2008) as well as Bayesian methods (Hutter, 2007), often in the context of array comparative genomic hybridization studies. Here, due to the density of the input dataset, we use regression trees to fit a piecewise constant function, although any segmentation algorithm able to handle hundreds of thousands of points could be used instead. Regression trees have been applied to WGS‐based detection of CNVs before (Chen et al., 2015), and they have been used in analysing variant‐level data from paired cancer samples (Putnam et al., 2017). We wrote the UN‐CNVc, a simple and fast CNV detection tool based on regression trees. Due to its sparse input format and the simplicity of the model used, it is able to process call sets from thousands of samples with WGS data in a reasonable time. A summary of the CNV calling pipeline is described in Figure 1a.

### 2.1 Identifying variant regions

Briefly, for each sample in 10Mb windows spanning the entire genome, we apply a regression tree using the rpart R library to the depth at marker sites normalised by chromosome‐wide depth. We use the default values of 0.01 for the complexity parameter of the regression tree (the overall *r* ^2^ of the model must increase of at least this value at each iteration) and 6 for the minimum leaf size. At sample sizes expected in cohort‐wide WGS data (>100) in 10Mbp windows, these parameters are very restrictive, that is, they will only fit a model that follows very broad variations of the data (Figure 1b). Assembling the constant segments of depth across the entire set of samples provides a global picture of broad depth changes in each 10Mb window (Figure 1c). Despite an apparent wide diversity of observed depths, the regressed segments cluster around multiples of 0.5 relative depth, as expected if these anomalies indeed corresponded to CNVs (Figure S2).

For each window, we fit a Gaussian mixture model, with means constrained to multiples of 0.5 within the observed depth range at that region. For each depth segment produced by the regression, we assign an ideal depth which is the multiple of 0.5 relative depth that is closest to the actual value of the segment. We also assign a score *s = 2p*, where *p* is the one‐sided *p* value for the Gaussian component centered around the ideal depth for that segment, and consider a call high‐quality when *s > 0.1*. We discretise the window in 5kb chunks, and consider a chunk as supporting a depth anomaly if the ratio of high‐quality versus low‐quality segments whose assigned depth is not 1 is greater than 1. This sets sensitivity to the highest level, guaranteeing that even a singleton is called as variable if a high‐quality segment is present. To determine boundaries, we then apply run‐length encoding to this variable, which produces regions in which a majority of high‐quality segments support a depth anomaly (Figure 1d). Application of this method on high‐depth WGS data suggests that duplications may exhibit more complex depth variations than deletions. We therefore also implement a deletion‐only mode, where only those segments that support deletions are used to call events.

### 2.2 Segment‐based genotyping

Because copy number events can be complex, it is common for a sample to have several segments, and hence several assigned depths per variable region. To produce a single genotype per individual, we compute the mean of the assigned depths weighted by the length of each segment, which is rounded to the next multiple of 0.5. Similarly, we produce an aggregate score summarising the average quality of the regressed segments for that sample. This allows for the easy application of a quality control (QC) step, whereby genotypes with too high a number of segments, or too low an aggregate quality can be set to missing.

### 2.3 Means‐based genotyping

The ability of the regression tree to correctly detect drops or increases in depth depends on the number of markers spanned by a CNV, as well as on the complexity parameter: for a constant complexity, smaller events are harder to distinguish from noise, hence harder to detect. At the limit of detection, it is, therefore, possible that not every carrier sample exhibits abnormal depth segments, leading to correct calling of the presence of a CNV, but false‐negative errors in genotyping. To address this issue, we implement means‐based genotyping, where each sample gets assigned the multiple of 0.5 that is closest to the average depth across all markers spanning the CNVs called by the regression step (Figure 1e). The quality score is then simply the distance between the average and assigned depths. This genotyping method is sensitive to the incorrect calling of CNV boundaries, but it can perform well on smaller events where segment‐based genotyping is inaccurate. We implement a manual genotyper, which applies means‐based genotyping on genomic coordinates specified by the user.

## 3 RESULTS

### 3.1 CNV calling in 6,898 European samples

We apply UN‐CNVc on WGS data from 6,898 samples across four studies: the MANOLIS and Pomak isolated cohorts from the HELIC study (Panoutsopoulou et al., 2014), the TEENAGE cohort of Greek adolescents (Ntalla et al., 2013), and the INTERVAL study of blood donors in the UK (Di Angelantonio et al., 2017). Similar sequencing protocol and identical SNV calling pipelines were used for the four cohorts to minimize batch effects (Supporting Information). A total of 401, 353, 349, and 973 CNVs were called from each cohort, respectively. A summary of sample sizes and quality metrics for each group is given in Table S1.

The genome was divided into 332 equal‐sized 10Mbp chunks, which were run in parallel, with some chunks empty due to overlap with pericentromeric regions. Runtime had a power dependency to sample size, between linear and quadratic (Figure S3a) with the linear model giving 2.4 s/sample (the best fit was for a $n^{1.5}$ dependency). On a cluster providing 332 threads, this means UN‐CNVc can call CNVs genome‐wide on a 1,000‐sample cohort in 40 min. Peak RAM usage was between a square and a cubic function of the sample number, with approximately 10Gb required for 3,000 samples (Figure S3b).

### 3.2 Quality control

QC of the variants was carried out based on the plots and statistics files generated by UN‐CNVc. Variants called within the centromeres and telomeres were first removed due to the low mapping quality in these regions. Following this, two rounds of QC were performed on the remaining CNVs. First, segment or boundary QC excluded variants based on calling metrics and diagnostics plots, with passing events having no multiple breaks within the call regions and homogenous boundaries (Figure S4). Second, genotype QC was performed using the genotype diagnostics plots. For complex events with multiple breakpoints, or small events with incorrect genotypes, boundaries were adjusted using the manual genotyper. (Figure S5).

**Figure 2:**
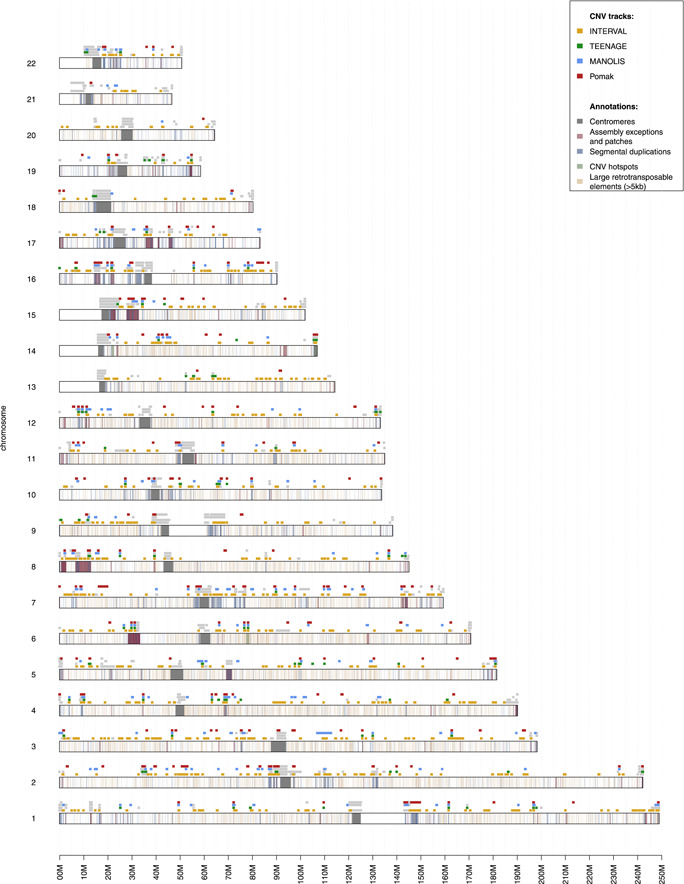
Chromosome map of all CNVs called by UN‐CNVc in four cohorts. Light grey tracks represent CNVs that failed QC, while the red, blue, green, and yellow tracks represent high‐quality CNVs in MANOLIS, Pomak, TEENAGE, and INTERVAL, respectively. Within the chromosomes, dark grey regions represent the centromeres. Regions marked in pink are assembly exceptions and patches, taken from the GRC data for GRCh38.p12, regions in blue are segmental duplications (from UCSC), regions in light green are “CNV hotspots,” which are known, highly variable regions comprising an intergenic region on chr6q14.1, an olfactory receptor gene cluster (*OR4C11‐OR5L2*) on chr11q11, a leukocyte immunoglobulin gene cluster (*LILRB3‐LILRB5*) on chr19q13.42, the immunoglobulin κ, λ, and heavy chain loci (*IGKC, IGLC1, IGH*), and the T cell receptor alpha locus (*TRA*). Regions in orange are large retrotransposable elements larger than 5kb, comprising Alus, SVAS, and L1, L2, and L3 elements. CNVs, copy number variants; QC, quality control; UN‐CNVc, Unimaginatively Named CNV caller

Following this QC procedure, we call 1,320 CNVs across the four cohorts (Table 1). Most of the variants that failed QC were concentrated within pericentromeric and telomeric regions (Figure 2). Assembly exceptions (stretches of DNA where genome assembly failed to produce a confident reference sequence) were particularly rich in CNVs, and although they tended to exhibit complex depth patterns, manual genotyping allowed to recover and genotype robust deletion signatures. Only a small minority (7.3%) of our high‐quality CNVs overlapped substantially (>50%) with segmental duplications and large retrotransposable elements (Table S2), which are highly variable regions prone to assembly errors. 101 (7.7%) high‐quality CNVs were shared between two or more cohorts, among which 12 were shared between all four cohorts and 37 between at least three cohorts. To make comparisons more meaningful, we applied a strict 80% reciprocal overlap criterion, which avoids counting as overlapping cases where a large event spans a much smaller one in another cohort. The largest overlap was between Pomak and INTERVAL, which shared 54 CNVs, followed by MANOLIS and INTERVAL, with 42 CNVs (Figure S6). As expected from their isolated nature, MANOLIS and Pomak exhibited a smaller proportion of singletons, doubletons, and rare CNVs compared to the cosmopolitan INTERVAL cohort (Figure S7).

**Table 1:** Number of CNVs called in each cohort

	MANOLIS	Pomak	TEENAGE	INTERVAL
Total called	401	353	349	973
Centromeric/telomeric regions	53	55	47	77
Failed regions (Figure S4b)	150	84	197	155
Deletions that passed both interval‐based QC and genotype QC	154	178	60	675
Regions that required manual genotyping	44	36	45	66
Manually genotyped deletions	58	50	49	96
Final no. of high‐quality deletions	212	228	109	771

*Note*: Interval‐based QC was done based on calling metrics and diagnostics plots, with passing events having no multiple breaks within the call regions and homogeneous boundaries, whereas genotype QC was performed using genotype diagnostics plots. Called events with inaccurate genotypes or complex regions containing multiple deletion events were manually genotyped. An example of a “failed region” is shown in Figure S4b. The final set of high‐quality deletion events comprises deletions passing both QC and the manually genotyped deletions.

Abbreviations: CNVs, copy number variants; QC, quality control.

CNVs were well tagged by SNVs, with 80%, 72%, 90%, and 84% of deletions having at least one SNV in high linkage disequilibrium (LD) (*r* ^2^ > 0.8) in MANOLIS, Pomak, TEENAGE, and INTERVAL, respectively (Table S3).

Cross‐population heterogeneities in allele frequencies are of particular interest when studying isolated populations such as the HELIC cohorts, due to the enhanced effects of genetic drift following the founder event. We compare the population deletion allele frequencies between any event that was present in at least two cohorts, adjusting for the number of comparisons performed ($p<\frac{0.05}{211}=2.37\times 10^{-4}$ for the two‐proportion *χ* ^2^ test). We find that 40.5% (41/101) of all shared deletions exhibit significant allelic frequency differences (Table S4). We only find modest common frequency differences in deletions shared between the HELIC isolates and the TEENAGE cohort, which is genetically closest, whereas most differences are found between the two Greek isolates and the UK‐based INTERVAL cohort. This is expected given the different ethnic background of the Greek and UK cohorts, as well as the lack of power to detect differences compared to the TEENAGE cohort due to its reduced sample size. The CNV showing the highest heterogeneity in frequency is the known 60kb esv3608493 deletion at 6p22.1, in a region containing four *HLA* pseudogenes, *HLA‐H, HLA‐T, HLA‐K*, and *HLA‐U*. The deletion occurs most frequently in MANOLIS (MAF = 0.2426) and TEENAGE (MAF = 0.2250), followed by Pomak (MAF = 0.1252) and then INTERVAL (MAF = 0.0987), with the most pronounced difference observed between MANOLIS and INTERVAL (*p *= 1.68x10^–80^). The low MAF of the variant in INTERVAL corresponds to findings from the 1000 Genomes Project Phase 3, where frequency in the GBR population was at 0.0879, lower than the European frequency of 0.1113.

### 3.3 Gene deletions

An average of 51% of our high‐quality deletions overlapped protein‐coding genes, with 45% of high‐quality events deleting at least one exon and 23% deleting one or more entire genes (Table S5). Some of these are common deletions that delete genes such as *RHD* and *GSTM1* (Supporting Information), while a number are in highly‐recombinant regions such as the immunoglobulin heavy chain locus on chromosome 14q32.33, and are unlikely to be functional. Additionally, we detect a known 58kb deletion overlapping the *BTNL8* and *BTNL3* genes that has been previously predicted to generate a fusion BTNL8/3 protein product (Aigner et al., 2013; Supporting Information). We also find evidence of known disease‐associated gene deletions in our cohorts, such as a common 30kb deletion of *APOBEC3B* (chr22:38982347‐38992804) that has been associated with increased risk of lung cancer, prostate cancer, (Gansmo et al., 2018), breast cancer, (Han et al., 2016; Long et al., 2013; Xuan et al., 2013) and HIV‐1 susceptibility (Singh et al., 2016), as well as a common CNV at the *FCGR3B* locus (1:161623196‐161631963) linked to autoimmune disease susceptibility (Fanciulli et al., 2007) and malaria severity (Faik et al., 2017).

### 3.4 Association analysis

In the MANOLIS cohort, 275 quantitative proteomic traits were assayed using the Proximity Extension Assay provided by Olink Proteomics across three protein panels (Cardiovascular II, Cardiovascular III, and Metabolism). We carried out association with the deletions called by UN‐CNVc using Plink 1.9. We also applied the linear mixed model implemented in GEMMA, where we accounted for relatedness using an empirical kinship matrix calculated on LD‐pruned common single‐nucleotide polymorphisms (SNPs) genome‐wide. Traits were transformed by applying the rank‐based inverse normal transformation, and adjusted for six covariates: sex, age, age‐squared, average levels across all proteins, season of the year, and assay plate. Four signals pass the genome‐wide significance threshold (*p *< 1.79x10^−6^ ≈  $\frac{0.05}{132\times 212}$, see Supporting Information). We examined signals down to a suggestive significance level of 1.0x10^−4^ (Table S6).

We detect deletion of the *NOMO1* gene (chr16:14833681‐14896160), associated with decreased NOMO1 protein levels (*β* = −0.6887, *σ* = 0.1323, *p* = 2.2x10^−7^). To account for potential genotyping error, the association was repeated using UN‐CNVc's raw estimates of mean depth as dosages instead of the assigned genotypes (*β* = −0.7841, *σ* = 0.1535, *p* = 3.75x10^−7^). There is no single‐point SNV association in that gene for NOMO1 protein levels. The closest SNV association is in the upstream *SHISA9* gene (rs200517050, *β *= −0.462, *σ* = 0.0855, *p* = 1.01x10^−7^), and a stronger association is also present in the *NOMO3* gene (rs3891245, intronic, *β* = −0.371, *σ* = 0.0476, *p* = 5.12x10^–14^). *NOMO1*, *NOMO2*, and *NOMO3* are closely located genes with very high sequence similarity (99.4% and 99.5% homology [BLAST]), and cannot be distinguished by the polyclonal antibody used in the OLINK proteomics assay. Both associations are independent, both of each other (*r* ^2^ < 1x10^−3^) and the deletion (*r* ^2^_rs200517050_ = 0.06, *r* ^2^_rs3891245_ = 1.2x10^−3^), suggesting that a *NOMO1* deletion and an intronic variant in *NOMO3* independently affect circulating levels of the NOMO proteins.

We find evidence of a complex CNV overlapping the *CCL3L3* gene and influencing CCL3 protein levels (Figure S8). We manually genotype a CNV (chr17:36195241‐36196130) affecting the last two exons of *CCL3L3*, which is associated with decreased CCL3 levels, both when assigned genotypes are used (MAF = 0.15, *β* = −0.378, *σ* = 0.05348, *p* = 2.55x10^–12^) and when raw mean depths are used (*β* = −0.4212, *σ* = 0.0573, *p* = 3.64x10^–13^). Copy‐number variation of *CCL3L3* and *CCL3L1*, its alias on an alternate haplotype (NT_187661.1) of chromosome 17, have been extensively studied. In addition to levels of their protein product (Townson, Barcellos, & Nibbs, 2002), they have been shown to be associated with rheumatoid arthritis (Ben Kilani et al., 2016; Nordang et al., 2012), immune reconstitution following HIV therapy (Aklillu et al., 2013), and protection against malaria (Carpenter, Farnert, Rooth, Armour, & Shaw, 2012). The gene product of *CCL3L3* binds to the same chemokine receptors as its close paralog *CCL3*, albeit with increased affinity, which suggests that the OLINK proteomics assay might not be able to differentiate the two ligands. This is even more likely as the two proteins are highly similar in sequence (95% homology; BLAST) and there is no commercially available antibody that can distinguish the two (Carpenter, McIntosh, Pleass, & Armour, 2012). Up to 14 copies of *CCL3L3* have been validated in some genomes (Sudmant et al., 2010), with the majority of people carrying 1–6 copies (Rimoin, Pyeritz, & Korf, 2013), whereas we confirm up to 7 copies in the MANOLIS cohort. It has been hypothesised that increased copy number of this gene resulted in higher levels of expression of its protein product, however in our study, including copy numbers greater than 2 in the model weakened the association compared to a deletion‐only model (Figure S9) suggesting that although deletion of *CCL3L3* decreases CCL3 levels, those levels are not affected by gene duplication.

## 4 DISCUSSION

### 4.1 Comparison with other callers

We compare UN‐CNVc's calling performance genome‐wide with PennCNV, an array‐based method, and the CNV discovery pipeline of GenomeSTRiP, a sequencing read‐based method, on 211 MANOLIS samples with both sequencing and CoreExome array data. On this subset, PennCNV took 2 hr to run with 586Mb peak RAM use, and GenomeSTRiP took 14.5 hr with peak RAM use of 3Gb, compared to 16 min and 798Mb for UN‐CNVc, excluding SNP calling using GATK3.5.

On these samples, UN‐CNVc calls 253 CNVs in deletion‐only mode, whereas PennCNV and GenomeSTRiP call 2,716 and 10,660 CNVs with minimum copy number <2, respectively. As expected, our method called on average larger CNVs than the other two methods (Figure S10). Fifty‐four (21%) of UN‐CNVc's events overlapped GenomeSTRiP's with 50% reciprocal overlap, however, 114 (45%) further regions called as variable by UN‐CNVc completely contained one or more GenomeSTRiP CNVs, and 127 (50%) were tagged (*r* ^2^ > 0.8) by at least one GenomeSTRiP CNV. Fifty‐seven (23%) of UN‐CNVc's CNV regions had 50% reciprocal overlap with the CNVs called by PennCNV, while a further 30 (12%) regions completely contained one or more PennCNV variants. The Database of Genomic Variants (DGV) is a repository of structural variants of >50 base pair (bp) curated from multiple large peer‐reviewed studies, including the 1000 Genomes Project. A higher percentage of CNVs detected by UN‐CNVc (155, 61%) had 50% reciprocal overlap with known CNVs in DGV (build 38, May 2016), compared to PennCNV 770 (29%) and GenomeSTRiP 3,384 (32%), although both methods called more known CNVs. By treating variants that can be found in DGV as true positives (TPs) and all other called variants as false positives (FPs), we calculate the precision (TP/TP+FP) and false discovery rates (FDRs; FP/TP+FP) of the three methods (Table S7). Our results show that despite its lower sensitivity UN‐CNVc offers the highest precision (61%) and the lowest FDR (39%) among the three methods.

### 4.2 FDR and novel events

In the full set of samples, we find an FDR of 18.1% on average across all four cohorts analysed (Table S8). Other measures such as specificity and sensitivity cannot be calculated with a database overlap method given that not all events present in such a database are expected to be present in analysed cohorts, preventing the calculation of a false negative rate. Conversely, this FDR is likely to be an overestimate, given that the DGV database was not built using data from these cohorts, and therefore might not include some TPs. To further assess the validity of such novel events, we also compare the UN‐CNVc callset to GenomeSTRiP in 211 samples for variants not present in the DGV database. A total of 253 deletion variants were called in 211 MANOLIS samples, of which 155 (61.3%) overlapped reciprocally with at least one variant in DGV by >50%. Of the remaining 98 deletions not found in DGV, three (3.1%) additional variants were also found in the GenomeSTRiP callset (50% reciprocal overlap). However, a further 17 (17.3%) UN‐CNVc regions completely contained at least one GenomeSTRiP variant in complete or high LD (*r* ^2^ > 0.8) with the corresponding UN‐CNVc deletion. This indicates that 20.4% of novel events can be validated using a third‐party in‐silico method.

### 4.3 Gene‐deleting regions

Sixty six (33%) of UN‐CNVc deletions affected entire genes, compared with 102 (4%) for PennCNV, and 74 (0.7%) for GenomeSTRiP. Of these, 56 (85%), 82 (80%), and 57 (77%), regions respectively have been previously reported (>50% reciprocal overlap) in DGV. Of the remaining 10 UN‐CNVc gene‐deleting regions not found in the DGV database, 4 regions overlap at least one GenomeSTRiP variant in high LD (*r* ^2 ^> 0.8). Notably, the complete deletion of the RHD gene was detected only by UN‐CNVc in the 211 MANOLIS samples. For the array‐based PennCNV, this was likely due to the lack of tagging SNPs within the region. Only five tagging SNPs in the CoreExome array were within the RHD gene coordinates, compared to 141 SNPs from the WGS data used by UN‐CNVc, demonstrating the advantage of using WGS data for CNV calling. For GenomeSTRiP, the deletion was split into six smaller CNVs with an average size of 11kb. This example, where the whole gene is known to be deleted, indicates that in some cases GenomeSTRiP may be tiling large CNVs by dividing them in smaller events.

Carrying on from this observation, we attempt to validate our gene‐deleting regions using GenomeSTRiP by calculating LD and genotyping concordance between our gene‐deleting events and any overlapping GenomeSTRiP events. Eighteen GenomeSTRiP regions containing 22 genes were in complete or high LD with their corresponding gene‐deleting UN‐CNVC events, with an overall genotyping concordance rate of 97.0% (Table S9). In the case of the RHD gene deletion, 4 out of 6 overlapping GenomeSTRiP variants were in complete LD with our event, 1 in high LD (*r* ^2^ = 0.980), and 1 in medium LD (*r* ^2^ = 0.532; Figure S11). This, along with clear patterns in the average depth across the region, suggests the presence of a large deletion that affects the entire gene, as well as several smaller deletions. This suggests that UN‐CNVc is much less sensitive to within‐population heterogeneity than GenomeSTRiP in complex regions where multiple CNVs are present.

### 4.4 Genotyping accuracy

Segment‐based genotyping tends to be biased towards the reference for smaller events, whereas means‐based genotyping is agnostic to variant size. Both methods should perform equally well for large variants. We calculate genotyping concordance for 54 CNVs that were called by both UN‐CNVc and GenomeSTRiP (defined as 50% reciprocal overlap; Table S10). The overall genotyping concordance and nonreference concordance rates were 96.1% and 85.9% for means‐based genotyping, and 79.3% and 22.3% for segment‐based genotyping, respectively (Supporting Information).

### 4.5 Limits of the piecewise constant regression model

Despite providing a certain level of automation, UN‐CNVc still requires post‐run manual QC, in the same way as array‐based genotypes require inspection of cluster plots. The software generates extensive diagnostic tables and plots to make this task easier for the user. Although piecewise constant regression can accurately model WGS depth in a single individual, UN‐CNVc leverages large sample sizes (*n* > 100) to differentiate signal from noise. Furthermore, since the software performs clustering on depth averages, a high enough depth (>15x) is required to ensure proper cluster separation. Finally, using marker‐level depth puts limits on the precision of the boundaries as well as the sizes of detected CNVs. The maximum precision achievable by a method such as UN‐CNVc is the distance between two consecutive SNVs; in practice, it is limited to around 10kb by the minimum leaf size, the segment aggregation algorithm and the discretization step (Table S11). Our method relies on at least one correct call by piecewise constant regression to genotype a CNV, which makes small, rare CNVs difficult to call.

For cases where the study design deviates from the ideal use case above, users can adjust the sensitivity of UN‐CNVc using several parameters. First, the complexity value passed directly to the regression tree directly influences the elasticity of the regression tree model (Figure S12). Smaller values allow the piecewise constant regression to follow depth more closely, therefore allowing to detect smaller CNVs but increasing the risk of FPs. This parameter can be adjusted by starting at the default value of 0.01 and decreasing it until a reference deletion (e.g. the *RHD* gene deletion) is correctly detected and the number of carriers stops increasing. Second, the window size, which should be increased from its default of 10 Mb if sample size is low (<100). Third, the ratio of high‐quality versus low‐quality segments required to call a deletion, which can be increased from its default value of 1 when analysing a particularly noisy depth signal. Fourth, the discretisation step, which is set by default at 5kb, and which determines the precision of the CNV boundaries. This value should not be smaller than the minimum distance separating two SNPs and should be kept reasonably large as decreasing it increases execution time linearly. In practice, changing parameters other than the complexity value should not be necessary under most use cases.

## 5 CONCLUSION

We demonstrate that it is possible to call large CNVs from variant‐level WGS depth information in large cohorts. Compared to other methods, UN‐CNVc performs well and offers better precision, although it is limited to large events. As a proof‐of‐concept, UN‐CNVc successfully detects well‐known deletions, such as the complete deletions of *RHD, GSTM1*, and *CCL3L1*, in 6,898 samples with deep WGS data. We conduct an association study with 272 quantitative protein levels in a set of 1,457 individuals and find two association signals, in which deletion of the *cis* gene caused a significant decrease in the resulting protein levels. These results provide proof of principle for cohort‐wide variant‐level depth approaches as a platform for discovering disease‐associated CNVs and genes. Although accurate read‐based methods that integrate within standard single‐nucleotide variant calling pipelines will remain the gold standard for CNV calling in terms of sensitivity, UN‐CNVc provides a computationally inexpensive means for CNV calling, using only the ubiquitously available per‐sample depth field from VCF files produced by widely‐used variant callers, including GATK (Van der Auwera et al., 2013), SAMtools (Li et al., 2009), and FreeBayes (Garrison & Marth, 2012). This approach is much less intensive than read‐based reanalysis and allows quick screening for areas harbouring copy number variation in cohorts where read‐level data is unavailable or intractable to process. With a classical approach, researchers wanting to analyse CNVs would analyse the reads twice: once for SNVs and once for CNVs. In the case of UN‐CNVc, calling is done without such an overhead when a SNV callset is present. Variable regions provided by our method can then be taken forward for read‐level analysis, which will provide bp resolution for breakpoints in CNVs of interest.

## CONFLICT OF INTERESTS

The authors declare that there are no conflict of interests.

## Supporting information

Supporting information

Supporting information

Supporting information

Supporting information

Supporting information

Supporting information

## Data Availability

The regression tree‐based approach, UN‐CNVc, is written in R and bash and is available on GitHub at https://github.com/agilly/un‐cnvc.
