# Supplementary material for: Population‐wide copy number variation calling using variant call format files from 6,898 individuals
Source: Genet Epidemiol. 2019 Sep 14;44(1):79–89. doi: 10.1002/gepi.22260 (PMC8653900; doi:10.1002/gepi.22260)
Supplement: Supplementary file 1 — Supporting information [file GEPI-44-79-s005.docx]

# Supplementary Data

# Supplementary Text

*Sequencing protocol and SNV Calling pipeline*

For MANOLIS, Pomak and INTERVAL, genomic DNA (500 ng) was sheared to a median insert size of 500 bp and subjected to standard Illumina paired-end DNA library construction. Adapter-ligated libraries were amplified by 6 cycles of PCR and subjected to DNA sequencing using the HiSeqX platform (Illumina) according to manufacturer’s instructions. For TEENAGE, one hundred samples from the general Greek population were sequenced, as well as the Genome in a Bottle NA12878 sample. Sample identity checks were performed using Fluidigm and aliquots prepared. These aliquots underwent library preparation using the standard HiSeqX method. Size selection was performed to target 350 base pairs. Sequencing was performed on the Sanger Institute’s Illumina HiSeqX plat-form with a target depth of 30x and PhiX spike-in.

Basecall files for each lane were transformed into unmapped BAMs using Illumina2BAM, marking adaptor contamination and decoding barcodes for removal into BAM tags. PhiX control reads were mapped using BWA Backtrack and were used to remove spatial artefacts. Reads were converted to FASTQ and aligned using BWA MEM 0.7.8 to the 1000 Genomes hg38 (GRCh38) with decoys (HS38DH) references. The alignment was then merged into the master sample BAM file using Illumina2BAM MergeAlign. PCR and optical duplicates are marked using biobambam markduplicates and the files were archived in CRAM format.

Per-lane CRAMs were retrieved and reads pooled on a per-sample basis across all lanes to produce library CRAMs; these were each divided in 200 chunks for parallelism. GVCFs were generated using HaplotypeCaller v.3.5 from the Genome Analysis Toolkit (GATK) for each chunk. All chunks were then merged at sample level, samples were then further combined in batches of 150 samples using GATK CombineGVCFs v.3.5. Variant calling was then performed on each batch using GATK GenotypeGVCFs v.3.5. The resulting variant callsets were then merged across all batches into a cohort-wide VCF file using bcftools concat.

Variant-level QC was performed using the Variant Quality Score Recalibration tool (VQSR) from the Genome Analysis Toolkit (GATK) v. 3.5, using a tranche threshold of 99.4% for MANOLIS and Pomak,, 99% for TEENAGE and INTERVAL. For INDELs, we used the recommended threshold of 1% in all cohorts.

*Complete gene deletions of* RHD *and* GSTM1

1. **RHD**

UN-CNVc detected a 70kb deletion spanning *RHD* (chr1:25272393-25330445) in the MANOLIS cohort, and we manually genotyped this known variant in other cohorts. The *RHD* gene codes for the rhesus D antigen (RhD), which determines an individual’s rhesus blood group. Rhesus-positive (Rh+) individuals express the antigen on the surface of their red blood cells, whereas rhesus-negative (Rh-) individuals do not. In Europeans, deletion of *RHD* is the most common cause of the Rh- phenotype(Flegel, 2007). We detected 122 (8%), 223 (14%), 15 (15%), and 888 (24%) homozygous individuals in MANOLIS, Pomak, TEENAGE, and INTERVAL, respectively. Only self-reported blood groups were available for some individuals in MANOLIS and Pomak, and as a result there was limited agreement between genotype and phenotype, where some individuals were Rh- despite having at least one copy of *RHD*.

| **MANOLIS** | Rh+ | Rh- |
| --- | --- | --- |
| Non-hom | 62 | 5 |
| Hom | 1 | 5 |

| **Pomak** | Rh+ | Rh- |
| --- | --- | --- |
| Non-hom | 70 | 12 |
| Hom | 1 | 10 |

1. **GSTM1**

Complete deletion of *GSTM1* (1:109682397-109702658) is a very common event, with an average allele frequency of 0.73 in Europeans (1000 Genomes Project Phase 3). The CNV was first detected by UN-CNVc in Pomak, following which we manually genotype the deletion in other cohorts. We find that for all of the cohorts, allele frequency was similar to the European average, at 0.73, 0.77, 0.72, and 0.73 for MANOLIS, Pomak, TEENAGE, and INTERVAL, respectively. GSTM1 null frequency was 53%, 61%, 55%, and 58% for the respective cohorts, which is within the 16%-60% range observed in past studies(Garte, et al., 2001; Kasthurinaidu, et al., 2015). The GSTM1 null genotype has been widely studied and associated with increased risk to a multitude of disorders, including, but not limited to hypertension (Petrovic and Peterlin, 2014), atherosclerosis (Grubisa, et al., 2018), Alzheimer’s disease (Wang, et al., 2016), and type 2 diabetes(Nath, et al., 2017).

1. **BTNL8, BTNL3**

The formation of chimeric genes is a common CNV consequence. We detect evidence for this in a 58kb deletion (chr5:180948042-181006042) that is shared among all four cohorts and deletes exons of two adjacent genes, *BTNL8* and *BTNL3.* The same deletion (chr5:180948027-181003596) was confirmed in a previous study to generate a fusion BTNL8/3 protein product which affected the expression of genes involved in immune response.

**a**


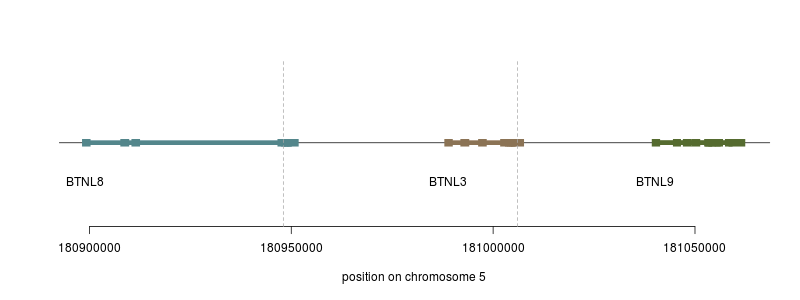


**b**


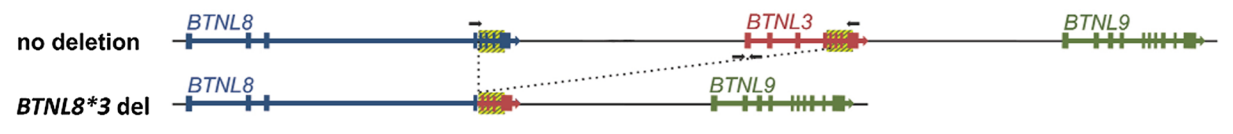


**Deletion spanning exons of *BTNL8* and *BTNL3* results in a *BTNL8/3* chimeric gene. (a)** Grey dotted lines denote the boundaries of the deletion called by UN-CNVc in all four cohorts. The deletion affects the last 4 exons of *BTNL8* and the first 7 exons of *BTNL3.* **(b)** Predicted formation of the *BTNL8/3* chimeric gene according to Aigner *et al.,* 2013(Aigner, et al., 2013). [Figure adapted from Aigner *et al.,* 2013]

*Study-wide significance threshold for association analysis with 275 quantitative proteomics traits*

We establish the study-wide significance threshold p <1.79x10^-6^ ≈ 0.05 / (132x212), where 132 is the effective number (Meff) of protein traits and 212 is the number of high-quality deletions in MANOLIS. Meff was calculated using Chevurud’s (2001) method(Cheverud, 2001).

*Genotyping concordance between UN-CNVc and other SV genotyping approaches*

A majority of discordant genotypes between means-based genotyping and GenomeSTRiP were attributed to a complex IGH region and a known event on chromosome 2 (UN-CNVc: chr2:34475308-34515308; GenomeSTRiP: chr2:34470737-34509725). For the latter, comparison of the MAFs (UNCNVc: 0.3571; GenomeSTRiP: 0.05476) with that of Europeans in 1000 Genomes Project Phase 3 (0.3956) suggests that UN-CNVc genotypes are more likely to be accurate.


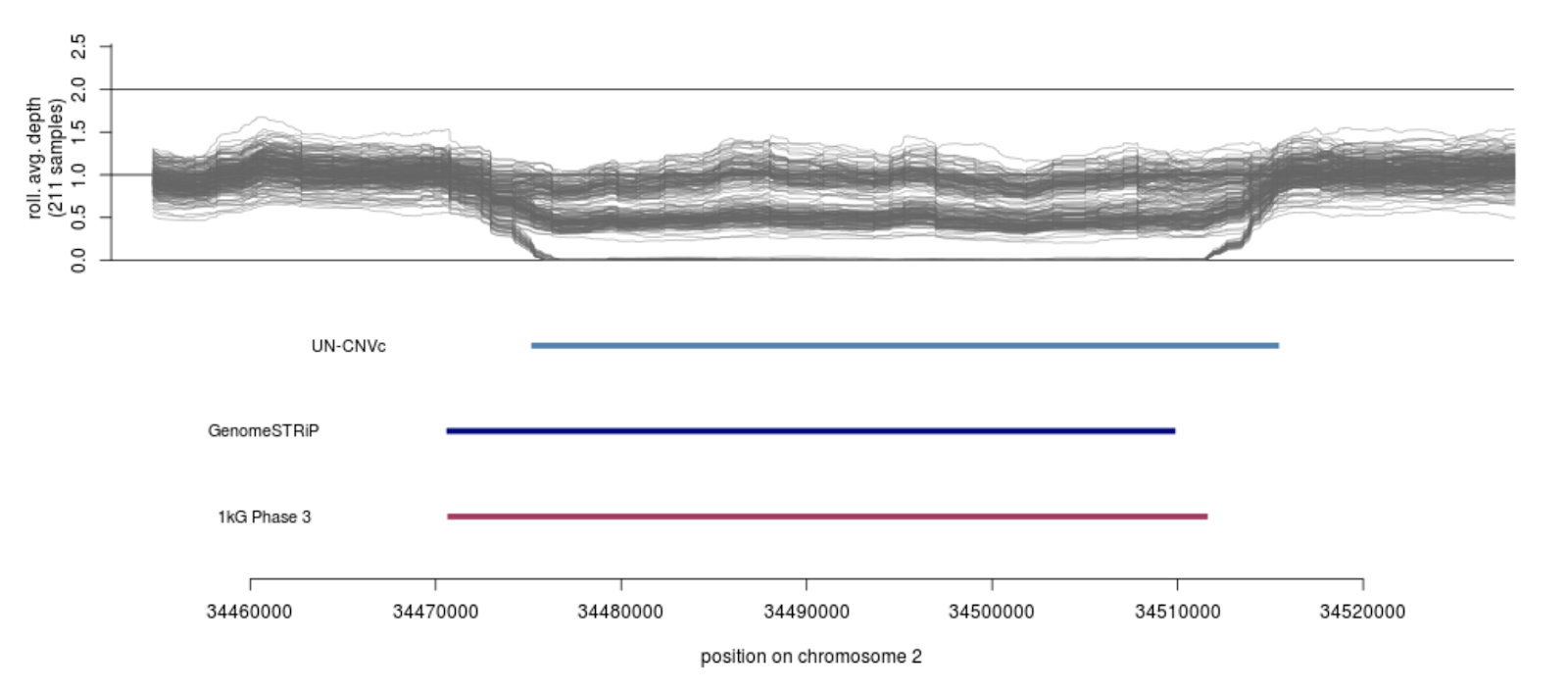


**Deletion region on chromosome 2 with discordant genotypes between UN-CNVc and GenomeSTRiP.** (Top) Rolling average depth at the chr2:34475308-34515308 region over 211 MANOLIS samples. (Bottom) Coordinates of the deleted regions detected by UN-CNVc (light blue), GenomeSTRiP (navy), and 1000 Genomes Phase 3 (maroon). No protein-coding genes are found in the region.

We further carry out genotyping concordance tests with other available SV genotyping methods; namely, DELLY and GenomeSTRiP’s SVGenotyper. Genotyping concordance for the common deletions of *RHD* (chr1:25266151-25334731; 1000 Genomes EUR MAF=0.3996) and *GSTM1* (chr1:109682397-109702658; 1000 Genomes EUR MAF=0.7227), as well as the complex CNV event at *CCL3L3* (chr17:36195241-36196130), were evaluated. For the two common deletions, complete concordance was observed between UN-CNVc and SVGgenotyper. DELLY performed similarly for *GSTM1*, but was unable to genotype the *RHD* deletion correctly (overall concordance=10.9%), calling the same number of individuals with the homozygous deletion as UN-CNVc, but only 4 individuals with the normal diploid copy number. For the complex *CCL3L3* event, however, concordance was low across the genotypers, with a score of 27.0% (non-reference concordance=8.5%) for SVGenotyper, and 34.1% (non-reference concordance=6.2%) for DELLY. A wide range of copy numbers from 0 to 7 was observed. These results suggest that genotyping accuracy of UN-CNVc is comparable to that of GenomeSTRiP’s and DELLY’s. For complex events with a wider range of copy numbers, however, further improvements to *in silico* SV genotyping methods are still required.

**Supplementary Tables**

**Supplementary Table 1: Sequencing characteristics for the four datasets analysed in this study.** For sequencing depth, standard deviations are given in parentheses.

|  | **MANOLIS** | **Pomak** | **TEENAGE** | **INTERVAL** |
| --- | --- | --- | --- | --- |
| **n** | 1,457 | 1,617 | 100 | 3,724 |
| **Mean depth (SD)** | 22.5 (3.53) | 18.4 (3.54) | 32.2 (2.61) | 18.4 (2.03) |
| **SNVs and INDELs** | 25,371,797 | 19,262,290 | 20,394,098 | 59,875,238 |

**Supplemental Table 2:** High quality deletions in overlapping segmental duplications and large retrotransposable elements. Large retrotransposable elements comprise Alus, SVAs, L1, L2, and L3 elements of size >5kb. A criterion of 50% reciprocal overlap was used.

|  | **MANOLIS** | **Pomak** | **TEENAGE** | **INTERVAL** |
| --- | --- | --- | --- | --- |
| **Total no. deletions** | 401 | 353 | 349 | 973 |
| **High-quality deletions** | 212 | 228 | 109 | 771 |
| **Segmental duplications** | 17 | 26 | 16 | 32 |
| **Large retrotransposable elements** | 3 | 1 | 1 | 0 |

**Supplementary Table 3 (separate file):** SNVs and INDELs showing the highest linkage disequilibrium among variants with r^2^>0.8. If several variants share the same r-squared with the same CNV, both are reported.

**Supplementary Table 4 (separate file):** Significant heterogeneities in frequencies of shared CNVs between cohorts. The significance threshold is p<2.16x10^-4^ ≈ 0.05/232, where 232 (10x6 + 24x3 + 50x2) is the number of pairwise comparisons performed.

**Supplementary Table 5. Gene-deleting regions in the four cohorts.**

|  | **MANOLIS** | **Pomak** | **TEENAGE** | **INTERVAL** |
| --- | --- | --- | --- | --- |
| **High-quality deletions** | 212 | 228 | 109 | 771 |
| **Gene-overlapping regions** | 116 | 121 | 43 | 397 |
| **Gene-deleting regions** | 52 | 60 | 22 | 172 |
| **Exon-deleting regions** | 105 | 106 | 38 | 349 |

**Supplementary Table 6. Suggestively significant associations in the MANOLIS cohort.** Study-wide significant associations are highlighted in green. Het and hom are the numbers of heterozygote and homozygote carriers in MANOLIS. BETA, SE and P are the effect size, standard error and p-value. For GEMMA, we report the likelihood ratio test p-value.

|  |  |  |  |  |  | **Plink 1.9** | | | **GEMMA 0.94** | | |
| --- | --- | --- | --- | --- | --- | --- | --- | --- | --- | --- | --- |
| **Protein** | **CHR** | **START** | **END** | **het** | **hom** | **BETA** | **SE** | **P** | **BETA** | **SE** | **P** |
| CCL3 | 17 | 36195241 | 36196130 | 364 | 39 | -0.3781 | 0.05348 | 2.56E-12 | -3.57E-01 | 5.49E-02 | 1.12E-10 |
| TFF3 | 6 | 29881000 | 29939000 | 549 | 79 | 0.2534 | 0.04561 | 3.35E-08 | 2.50E-01 | 4.69E-02 | 1.47E-07 |
| NOMO1 | 16 | 14833681 | 14896160 | 62 | 1 | -0.6887 | 0.1323 | 2.24E-07 | -7.15E-01 | 1.39E-01 | 3.31E-07 |
| TNFR1 | 6 | 31251000 | 31262000 | 155 | 4 | -0.4003 | 0.08286 | 1.51E-06 | -3.48E-01 | 9.05E-02 | 1.33E-04 |
| PPP1R2 | 7 | 53390000 | 53525000 | 74 | 3 | -0.5636 | 0.1182 | 2.06E-06 | -4.68E-01 | 1.23E-01 | 1.64E-04 |
| TYMP | 7 | 53390000 | 53525000 | 74 | 3 | -0.5382 | 0.113 | 2.10E-06 | -4.80E-01 | 1.19E-01 | 6.54E-05 |
| CD1C | 6 | 31269000 | 31271000 | 594 | 760 | 0.1984 | 0.04437 | 8.43E-06 | 1.91E-01 | 4.70E-02 | 5.62E-05 |
| CA13 | 5 | 180948000 | 181005000 | 583 | 125 | 0.1893 | 0.04279 | 1.06E-05 | 1.70E-01 | 4.39E-02 | 1.18E-04 |
| NADK | 7 | 53390000 | 53525000 | 74 | 3 | -0.5019 | 0.1139 | 1.14E-05 | -4.25E-01 | 1.20E-01 | 4.48E-04 |
| PSPD | 10 | 797150000 | 79747000 | 681 | 249 | -0.1717 | 0.03905 | 1.19E-05 | -1.91E-01 | 4.10E-02 | 3.24E-06 |
| CCL24 | 7 | 40205016 | 40295016 | 4 | 0 | 2.514 | 0.5748 | 1.31E-05 | 2.25E+00 | 6.97E-01 | 1.35E-03 |
| NEMO | 7 | 53390000 | 53525000 | 74 | 3 | -0.4965 | 0.1154 | 1.80E-05 | -4.35E-01 | 1.21E-01 | 3.54E-04 |
| MB | 8 | 16080068 | 16150000 | 18 | 0 | -1.148 | 0.268 | 1.96E-05 | -1.12 | 2.66E-01 | 2.93E-05 |
| UPAR | 7 | 53390000 | 53525000 | 74 | 3 | -0.4818 | 0.1132 | 2.24E-05 | -3.86E-01 | 1.20E-01 | 1.42E-03 |
| CDHR5 | 16 | 76920071 | 77015071 | 8 | 0 | 1.581 | 0.3753 | 2.69E-05 | 1.12E+00 | 4.01E-01 | 5.26E-03 |
| CTSL1 | 2 | 84110191 | 84185191 | 12 | 0 | -1.203 | 0.2881 | 3.19E-05 | -1.15 | 3.10E-01 | 2.47E-04 |
| UPAR | 6 | 31251000 | 31262000 | 155 | 4 | -0.3444 | 0.08315 | 3.67E-05 | -3.11E-01 | 9.04E-02 | 6.11E-04 |
| TM | 6 | 29881000 | 29939000 | 549 | 79 | 0.1912 | 0.0462 | 3.71E-05 | 1.90E-01 | 4.93E-02 | 1.23E-04 |
| PAG1 | 7 | 53390000 | 53525000 | 74 | 3 | -0.4732 | 0.1143 | 3.72E-05 | -4.23E-01 | 1.20E-01 | 4.67E-04 |
| CD164 | 5 | 180948000 | 181005000 | 583 | 125 | 0.1749 | 0.04235 | 3.86E-05 | 1.38E-01 | 4.39E-02 | 1.75E-03 |
| ST2 | 22 | 25245222 | 25560222 | 16 | 0 | -1.145 | 0.2778 | 3.98E-05 | -9.89E-01 | 2.87E-01 | 6.18E-04 |
| GRAP2 | 7 | 53390000 | 53525000 | 74 | 3 | -0.6167 | 0.15 | 4.29E-05 | -5.36E-01 | 1.52E-01 | 4.72E-04 |
| SERPINB6 | 6 | 31251000 | 31262000 | 155 | 4 | -0.3349 | 0.08286 | 5.62E-05 | -2.93E-01 | 8.97E-02 | 1.13E-03 |
| CCL15 | 12 | 17500025 | 17650025 | 4 | 0 | 2.024 | 0.5007 | 5.62E-05 | 1.78E+00 | 5.31E-01 | 8.80E-04 |
| IL27 | 8 | 2190000 | 2270000 | 90 | 1 | -0.4441 | 0.11 | 5.74E-05 | -4.32E-01 | 1.20E-01 | 3.54E-04 |
| PILRB | 6 | 31251000 | 31262000 | 155 | 4 | -0.334 | 0.08291 | 5.95E-05 | -3.08E-01 | 9.21E-02 | 8.61E-04 |
| CCDC80 | 7 | 53390000 | 53525000 | 74 | 3 | -0.4539 | 0.1139 | 7.08E-05 | -3.48E-01 | 1.20E-01 | 4.29E-03 |
| FCRL1 | 6 | 103290023 | 103315500 | 724 | 410 | 0.1546 | 0.03883 | 7.26E-05 | 1.10E-01 | 4.05E-02 | 7.15E-03 |
| IL18 | 6 | 73885313 | 73892900 | 578 | 122 | -0.1688 | 0.04278 | 8.37E-05 | -1.75E-01 | 4.43E-02 | 8.24E-05 |
| PRTN3 | 7 | 8790064 | 8830064 | 127 | 4 | 0.3607 | 0.09182 | 9.02E-05 | 2.75E-01 | 9.80E-02 | 5.28E-03 |
| ITGB2 | 7 | 8790064 | 8830064 | 127 | 4 | 0.3605 | 0.09184 | 9.10E-05 | 3.11E-01 | 9.64E-02 | 1.46E-03 |
| MMP9 | 6 | 31251000 | 31262000 | 155 | 4 | -0.3235 | 0.08282 | 9.84E-05 | -2.88E-01 | 9.13E-02 | 1.70E-03 |

**Supplementary Table 7: Precision and FDR comparison between UN-CNVc, PennCNV, and GenomeSTRiP.** Variants overlapping by at least 50% reciprocally with at least one variant in DGV were considered True Positives (TP), while variants not found in DGV were considered False Positives (FP). Precision and FDR were calculated according to the formulas TP/(TP+FP) and FP/(TP+FP), respectively.

|  | **Total called** | **Overlap (n)** | **Precision (%)** | **FDR (%)** |
| --- | --- | --- | --- | --- |
| **UN-CNVc** | 253 | 155 | 61.3 | 38.7 |
| **PennCNV** | 2,716 | 770 | 28.4 | 71.7 |
| **GenomeSTRiP** | 10,660 | 3,384 | 31.7 | 68.3 |

**Supplementary Table 8: Precision and FDR of UN-CNVc in four cohorts.** High quality variants overlapping by at least 50% reciprocally with at least one variant in DGV were considered True Positives (TP), while variants not found in DGV were considered False Positives (FP). Precision and FDR were calculated according to the formulas TP/(TP+FP) and FP/(TP+FP), respectively.

|  | **High quality deletions** | **Overlap (n)** | **Precision (%)** | **FDR (%)** |
| --- | --- | --- | --- | --- |
| **MANOLIS** | 212 | 168 | 79.2 | 20.8 |
| **Pomak** | 228 | 186 | 81.6 | 18.4 |
| **TEENAGE** | 109 | 94 | 86.2 | 13.8 |
| **INTERVAL** | 771 | 621 | 80.5 | 19.5 |
| **Total** | 1,320 | 1,069 | 81.9 | 18.1 |

**Supplementary Table 9 (separate file):** Linkage disequilibrium and genotyping concordance of gene deleting events between UN-CNVc and GenomeSTRiP.

**Supplementary Table 10 (separate file):** Genotyping concordance for common CNVs called by both UN-CNVc and GenomeSTRiP. Common CNVs were defined as events that overlapped by at least 50% reciprocally.

**Supplementary Table 11 (separate file):** Breakpoint precision of high-quality deletion events in MANOLIS, Pomak, TEENAGE, and INTERVAL. Precision was calculated by adding the distances to the nearest SNP on the left and right of each breakpoint.

**Supplementary Figures**

**Supplementary Figure 1: Local variations in depth averaged at variant sites in different window sizes.** Window sizes are in base pairs. Standard deviations are expressed in units of coverage, and are computed on chromosome 11 for one MANOLIS individual. The rightmost point corresponds to the recommended window size of UN-CNVc.

**
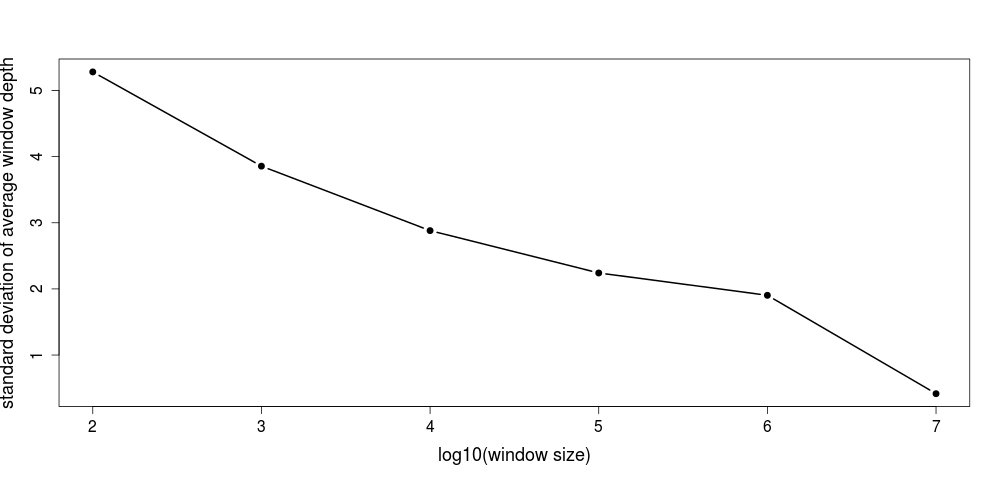
**

**Supplementary Figure 2: Depth segments after piecewise constant regression prior to QC.** Segment depths are counted for all CNVs called in the HELIC MANOLIS cohort in deletion-only mode. Segments with a called depth of 1 are masked due to their number, so as not to eclipse other depths. Note the clear peaks around 0 and 0.5 for depth segments. Duplication signatures clustering around expected multiples of 0.5 is less apparent, as expected in deletion-only mode.


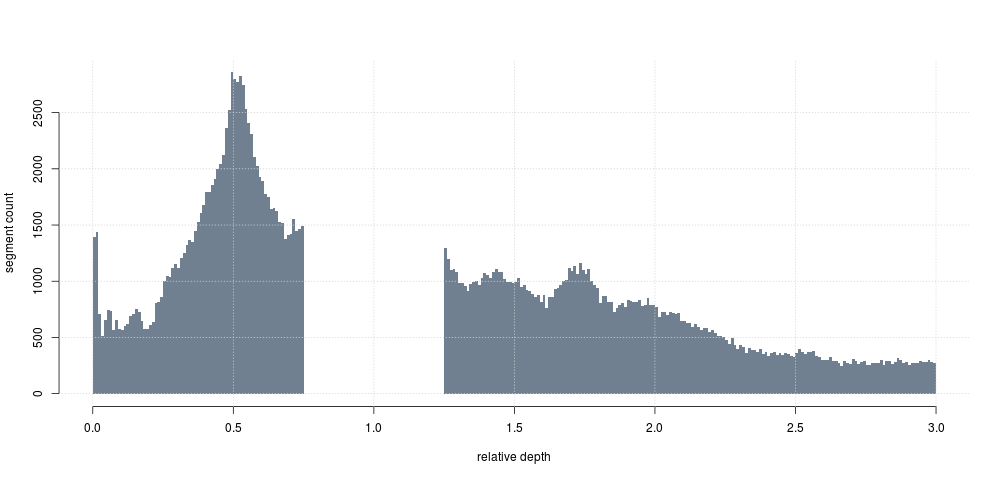


**Supplementary Figure 3: a.** Median runtime as a function of sample size for all three cohorts analysed as part of this study. The black line is a linear model fitted to the data with the intercept constrained to 0. Dashed lines represent the ten$y=x^{1+0.1n}$(n natural>1) power functions between the linear and quadratic models. Red lines represent intervals of one median absolute deviation (MAD) around the median. **B.** Peak RAM usage. Dashed lines represent the four first$y=x^{1+0.5n}$functions (n natural>1).


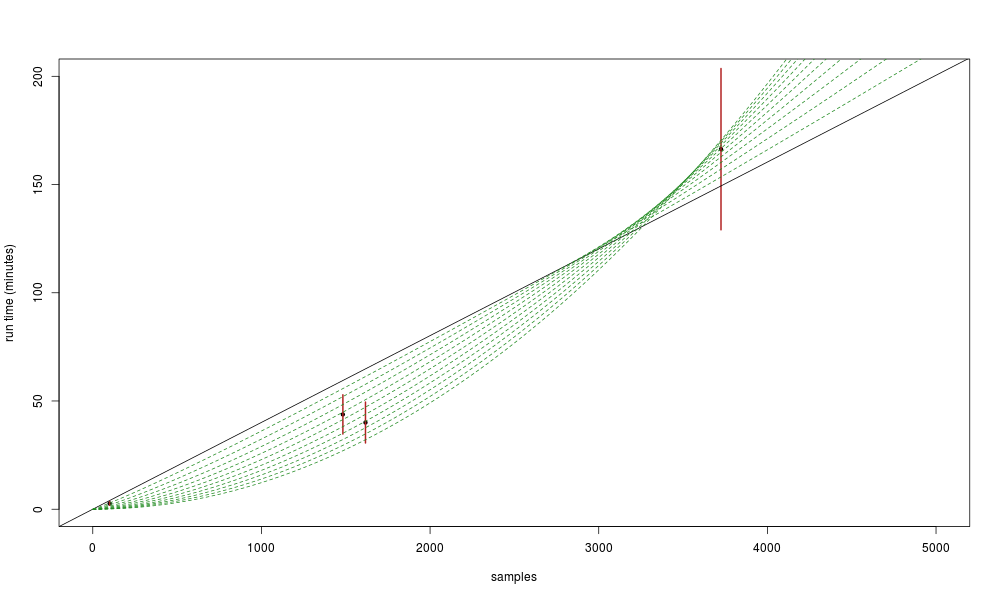
**a.**


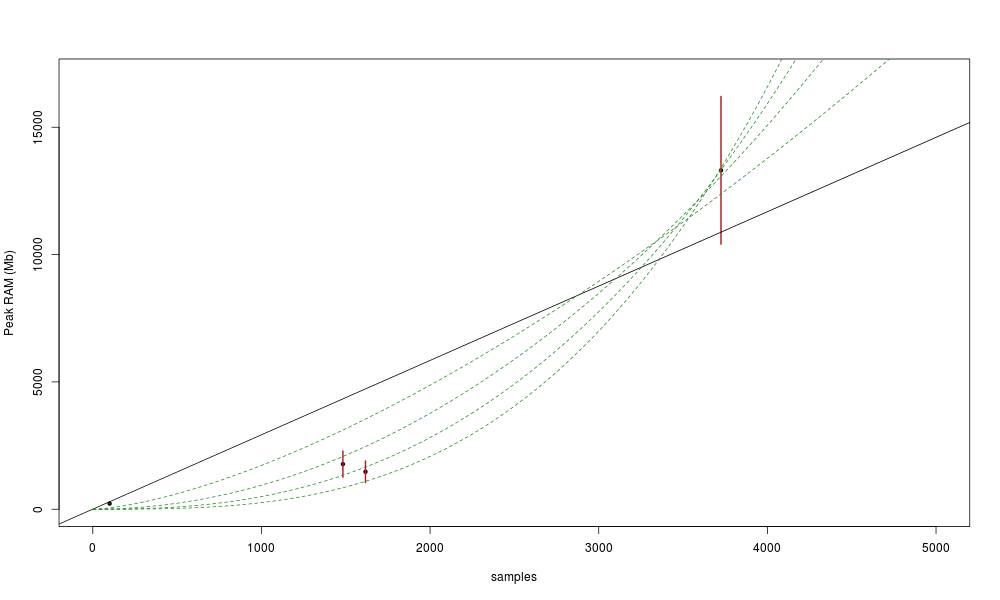
**b.**


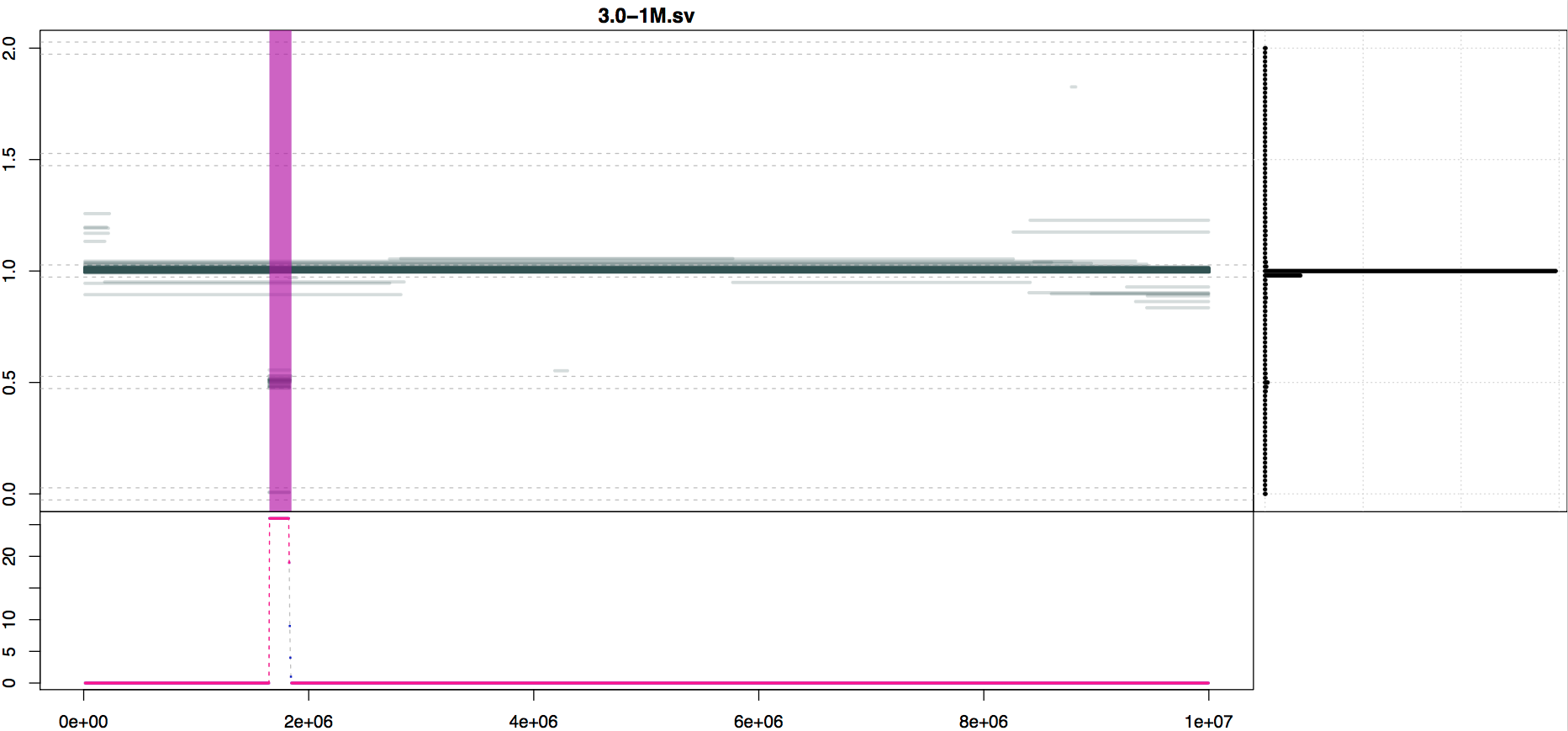
**Supplementary Figure 4: a.** Diagnostic plot of a high-quality and **b.** low-quality deletion based on boundary QC. The top left panel shows the called depth segments in the region with the regions called by RLE in purple. The right panel shows the histogram of weighted segment depths used to fit the Gaussian model. The bottom panel shows the count and RLE graph over the region.

**a.**

**b.**


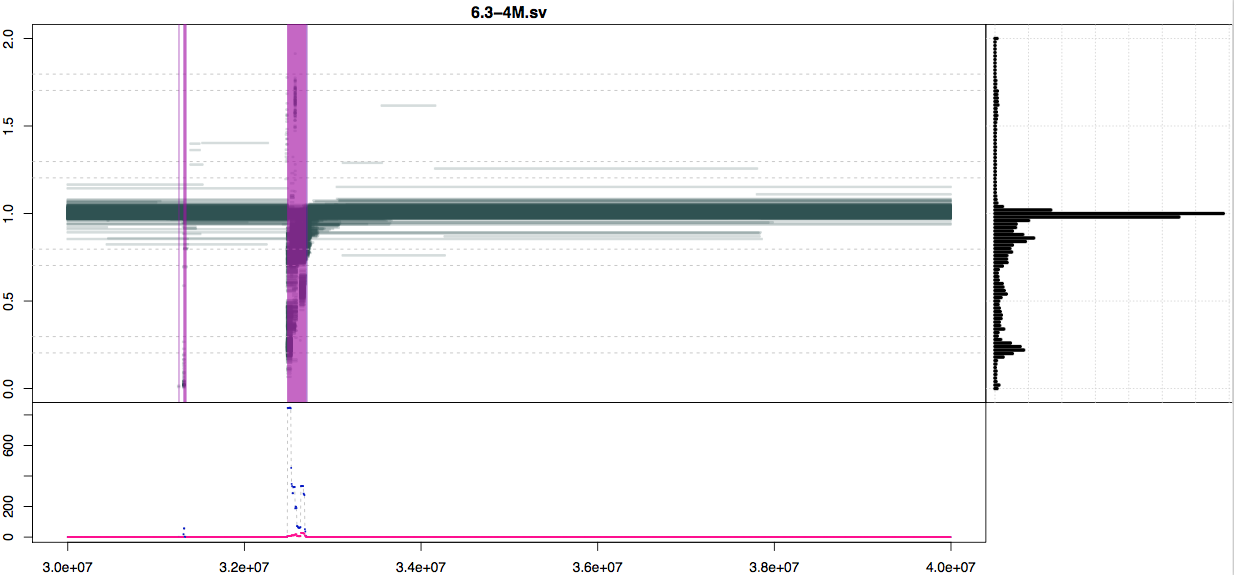
**Supplementary Figure 5: a.** Diagnostic plots of a CNV that requires manual genotyping, based on genotype QC. Top left: average marker depth over called region. Top right: rolling average mean (*k=l/10* where *l* is the length of the called region) for every sample in the region. Bottom right: segments called in the region, with deletion-carrying samples in red. Bottom left: segment (y axis, colour indicates called genotype) and means (x axis, symbol indicates genotype)-based genotype in the region. Means-based genotyping performs better, but overestimates depth for homozygotes. In this example, different segment boundaries have been called in different samples, leading to an overestimation of the deletion size. This has the consequence of biasing depth estimates upwards (top left) with homozygotes incorrectly called as heterozygotes. **b.** Diagnostic plots after manual genotyping. The boundaries and genotypes have been corrected.


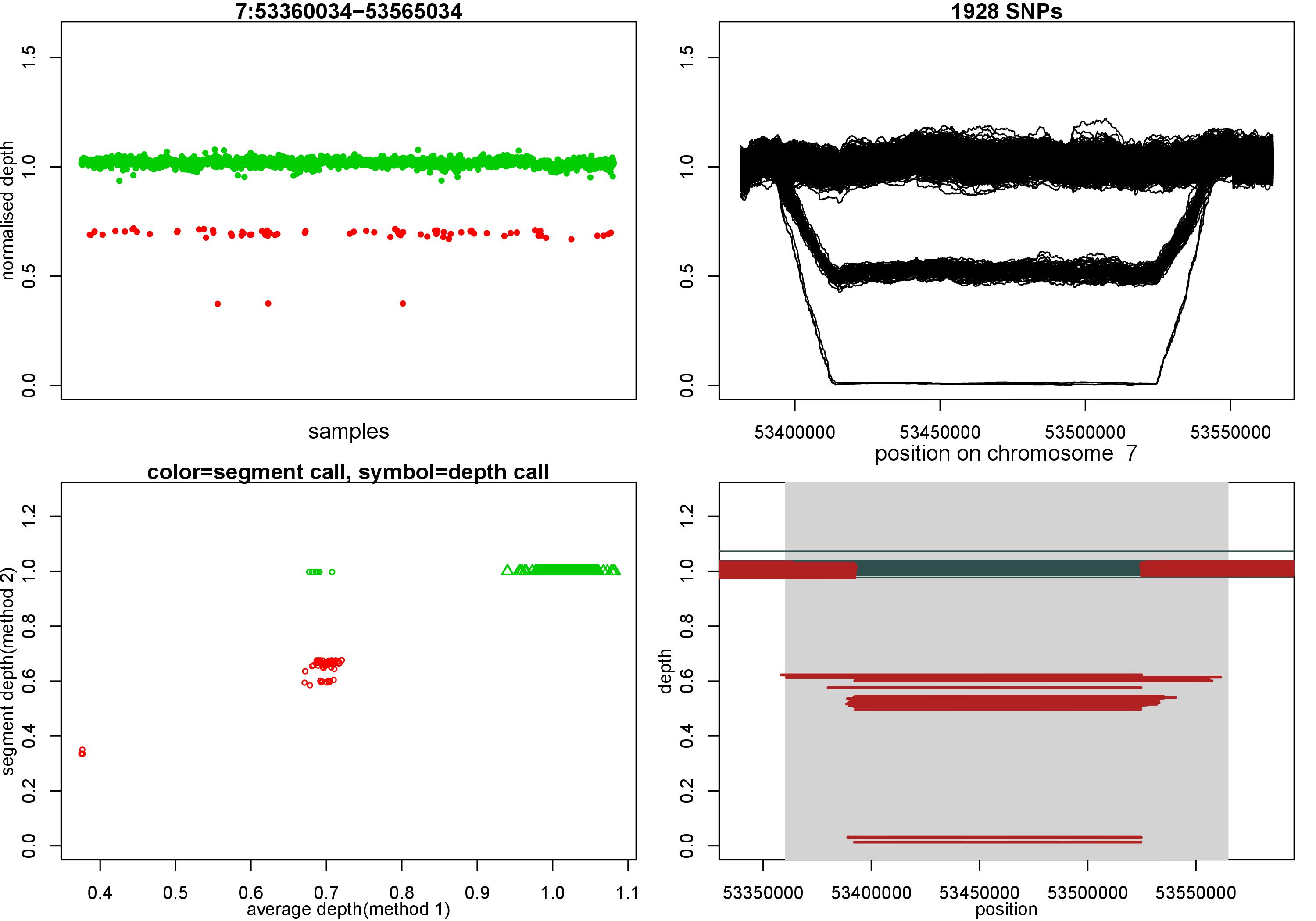
**a.**

**
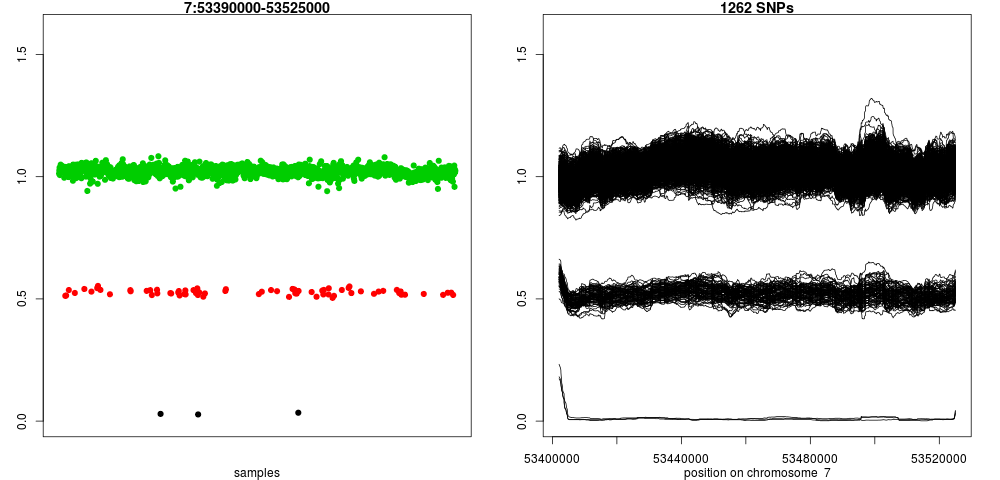
b.**

**Supplementary Figure 6:** Overlap between the four cohorts after segment-based and genotype quality control.

**
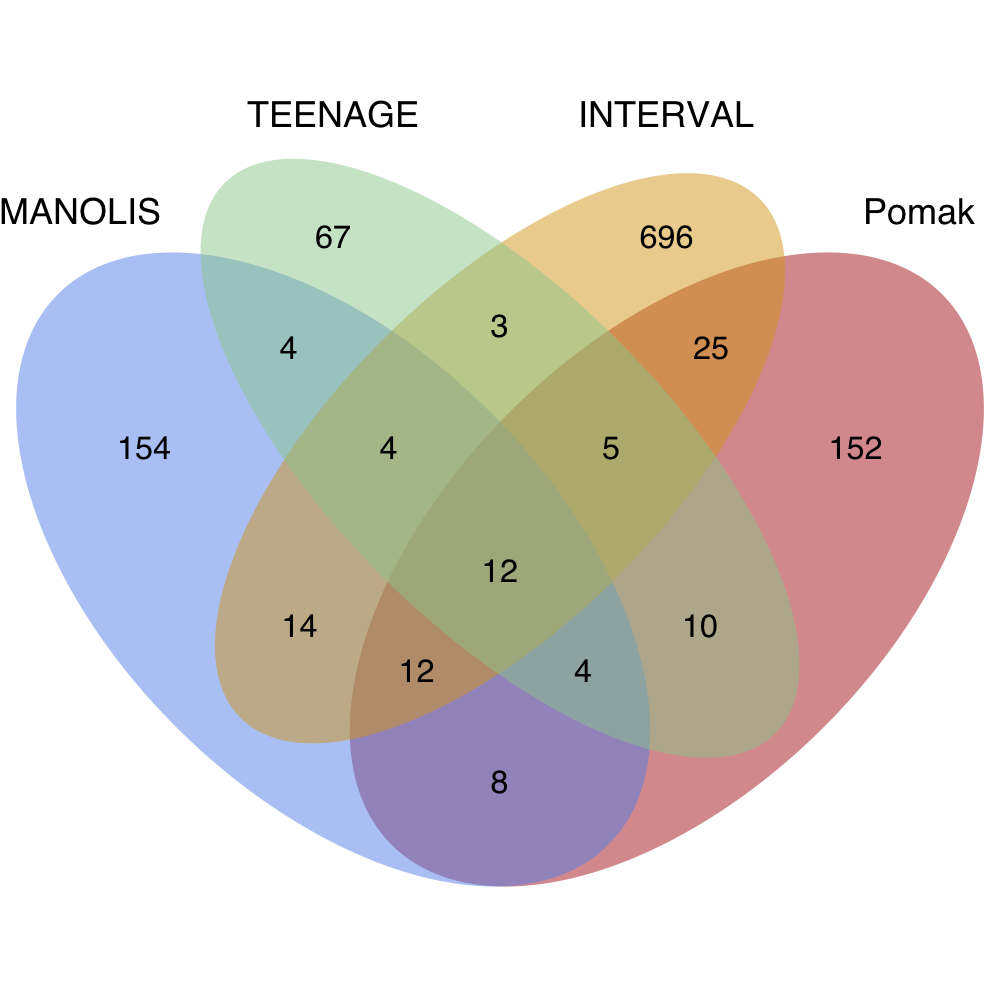
**

**Supplementary Figure 7:** Comparison of CNV frequency makeup between MANOLIS, Pomak and INTERVAL. The bars denote 95% binomial proportion confidence intervals. TEENAGE is not represented due to its sample size, which is one order of magnitude smaller, enough to influence the sensitivity of UN-CNVc.


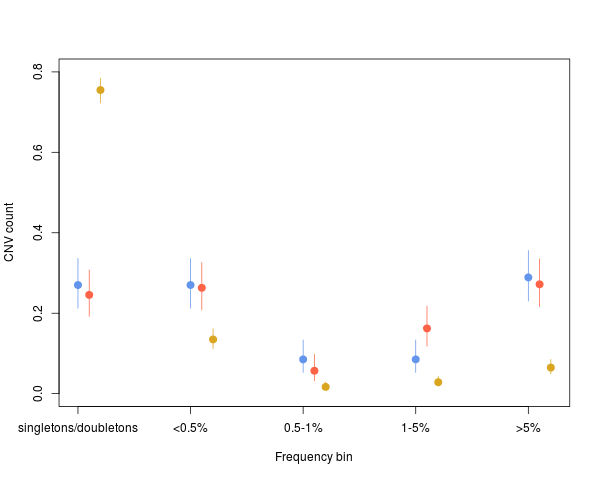


**Supplementary Figure 8:** *CCL3* region and read depth. The dashed vertical line is the left boundary of the CNV called by UN-CNVc, which extends to the right of the displayed window. The shaded blue region is the CNV which was manually genotyped and gave rise to the association. Top plot: Red lines are the regressed depth segments belonging to individuals carrying a deletion of *CCL3L3*. The deletion is not localised to *CCL3L3* but extends far downstream, across a SNP-poor region. The duplication of *CCL3L3* by contrast is a much smaller event, which we used for determining the boundaries of the region to genotype. Protein coding genes and assembly exceptions are annotated below the plot. Second plot from top: Rolling average depth across 100 randomly selected samples confirms the complexity of this region. The *CCL4-TBC1D3B* duplication, the *CCL3L3* duplication and the *CCL3L3-TBC1D3F* deletion seem to be distinct events with differing depth patterns. The SNP-poor region downstream of *CCL3L3* also has lower average depth and SNP density, indicating a potentially difficult to access segment or a reference issue (third from top plot). This paucity of variants is reflected on the single-point association plot (bottom plot).The top associated variant is in strong LD with known *CCL3* pQTLs, and tags the deletion. (Figure on next page)


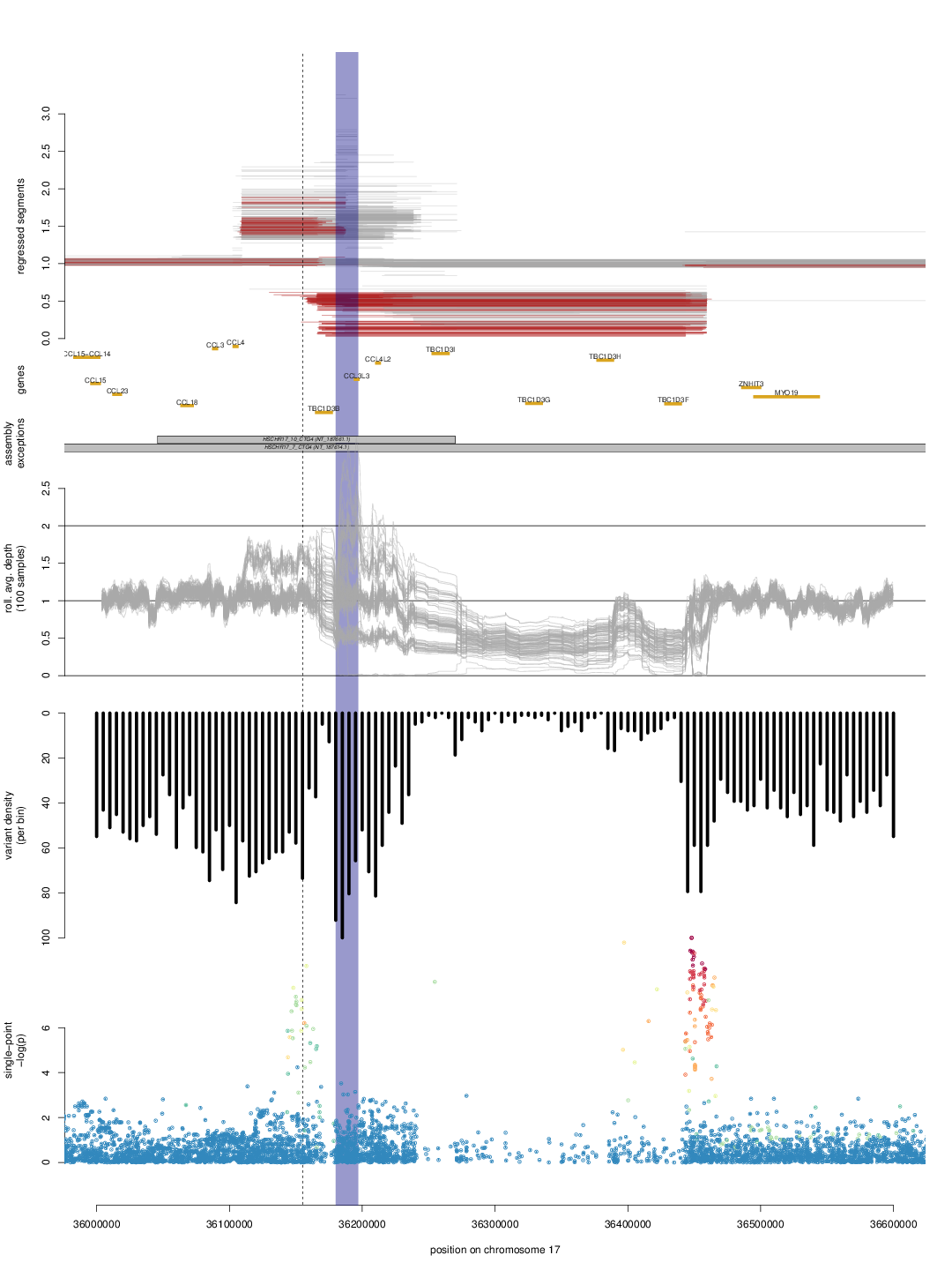


**Supplementary Figure 9:** Association of *CCL3L3* deletion with CCL3 protein levels under the deletion and duplication (top) and deletion only (bottom) models. ANOVA are applied to called copy numbers, linear models are applied to uncalled relative depth.


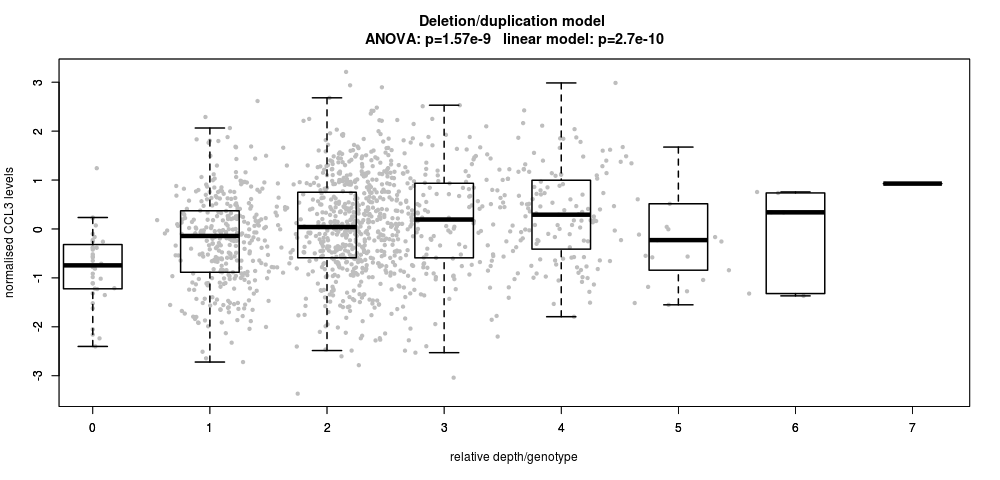


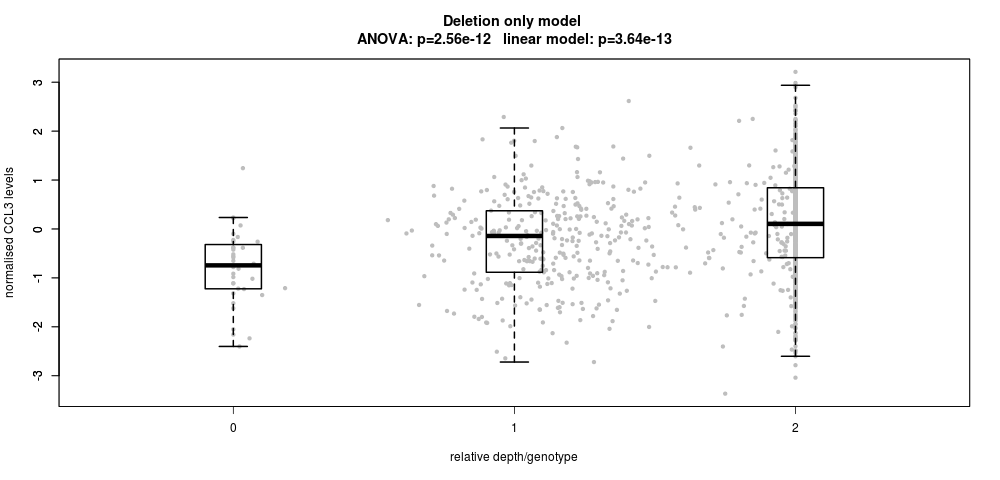


**Supplementary Figure 10:** Base 10 logarithm of the sizes of the CNVs called by UN-CNVc, GenomeStrip, and PennCNV.


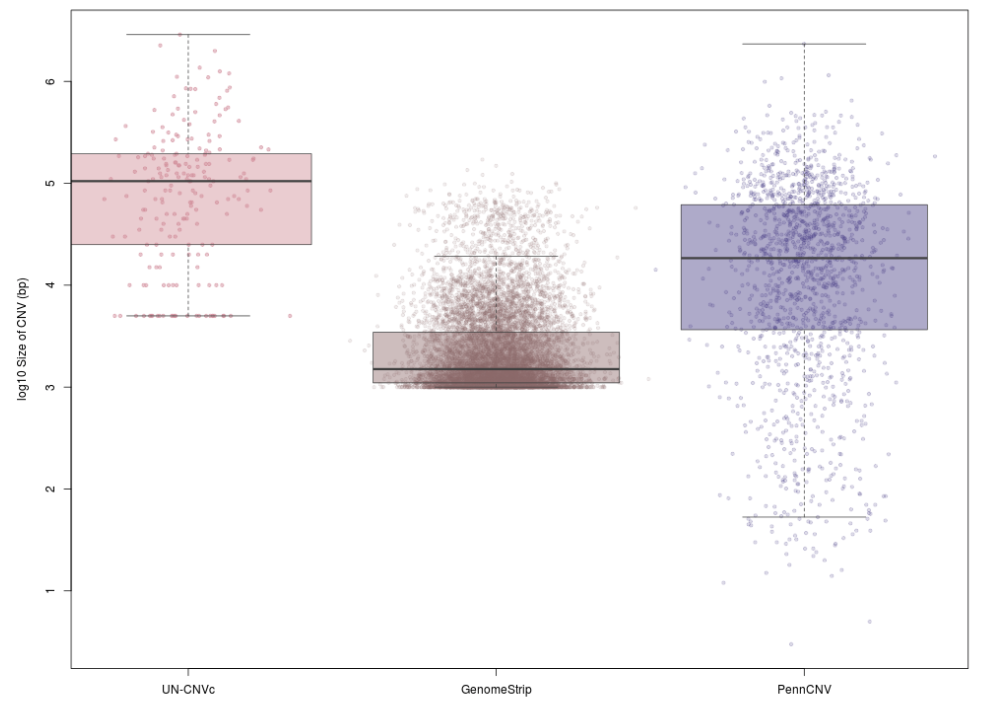


**Supplementary Figure 11: Depth and deletions in the RHD region.** Top plot: Rolling average depth across the region for 100 MANOLIS samples. Bottom plot: Deletion events called by UN-CNVc (light blue) and GenomeSTRiP (navy blue and grey) and the RHD gene. GenomeSTRiP events in complete or high (r^2^>0.8) LD with the UN-CNVc event are in navy blue, while events not in LD are in grey. UN-CNVc calls only one deletion spanning the RHD gene, while GenomeSTRiP calls 6 smaller overlapping deletions.


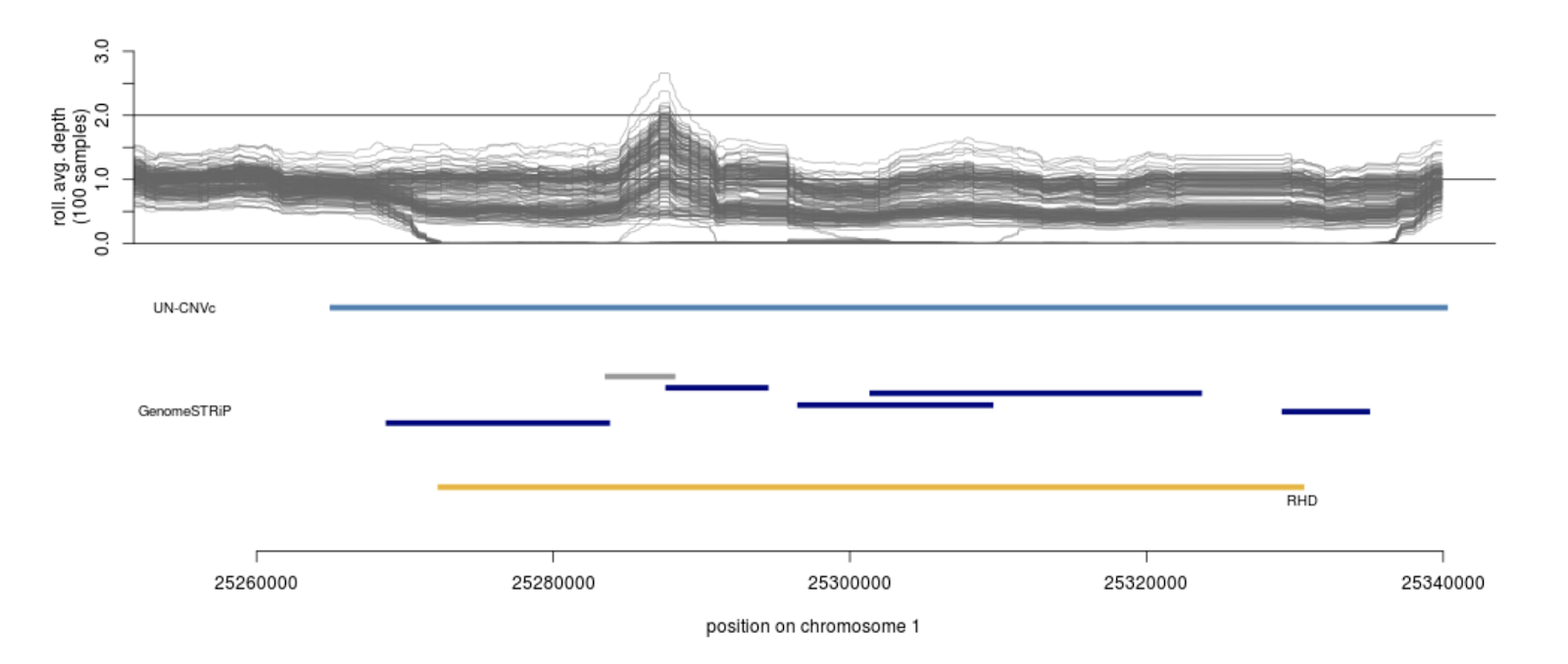


**Supplementary Figure 12: Influence of the complexity parameter *c* on depth regression.**  Tested values of *c* are 1x10^-4^ for **a.**, 3x10^-4^ for **b.**, and 5x10-4 for **c.** The default used by UN-CNVc is the default of 0.01, with an option allowing the user to specify more sensitive values. Depth is measured on chromosome 11 in the Pomak cohort.


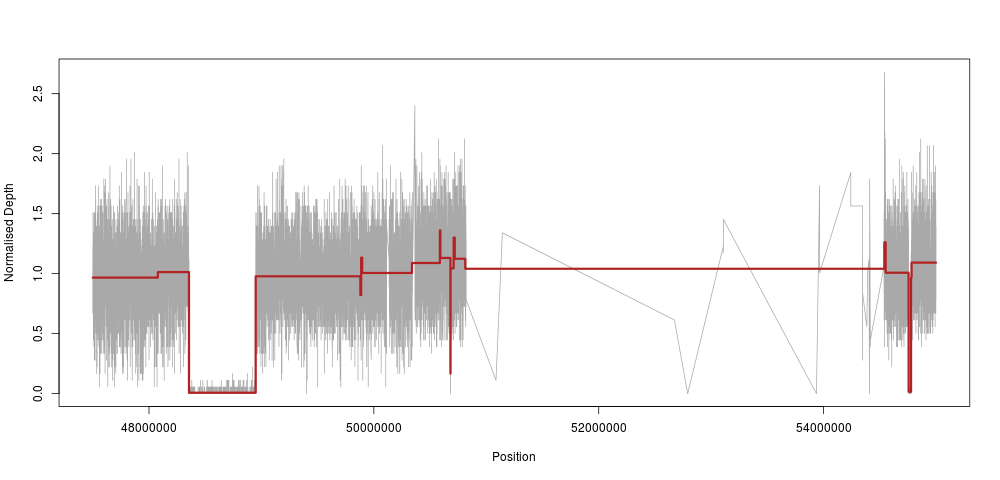

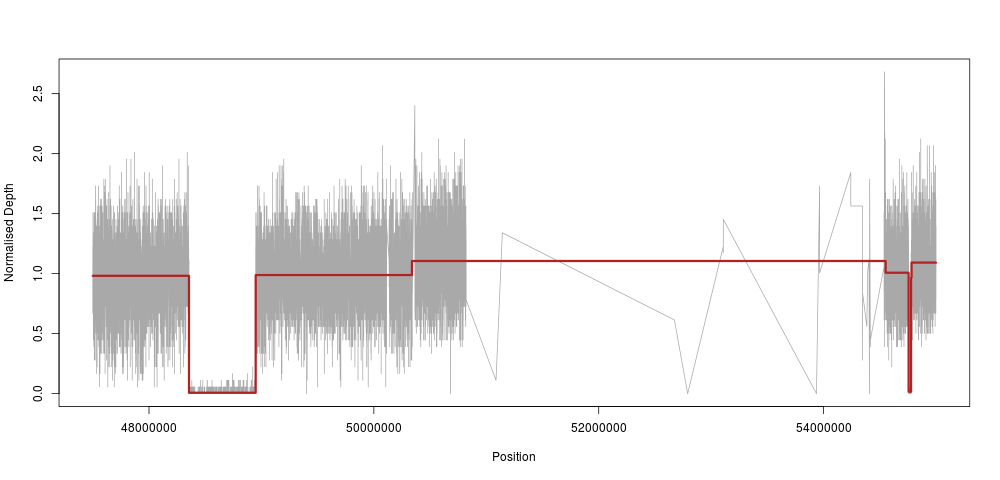
**a.**
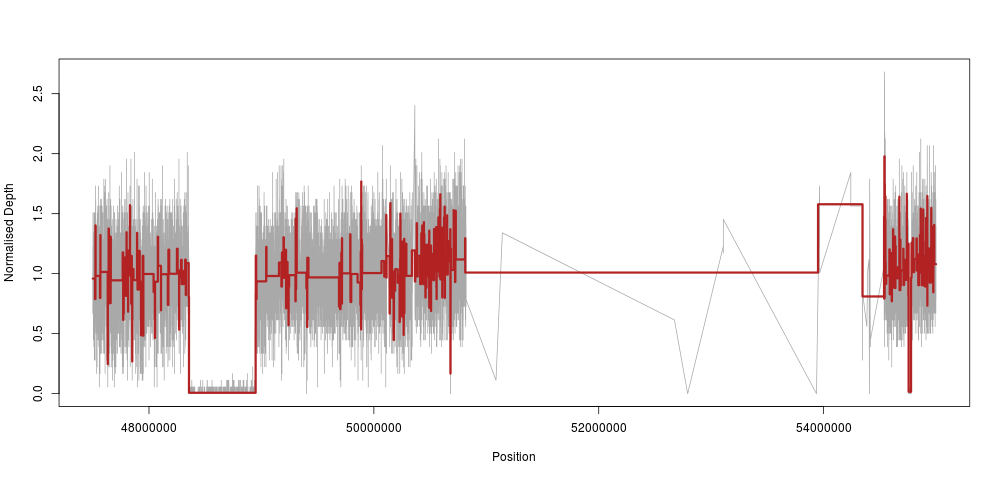


**c.**

**b.**

**References**

Aigner, J.*, et al.* A common 56-kilobase deletion in a primate-specific segmental duplication creates a novel butyrophilin-like protein. *BMC Genet* 2013;14:61.

Cheverud, J.M. A simple correction for multiple comparisons in interval mapping genome scans. *Heredity (Edinb)* 2001;87(Pt 1):52-58.

Flegel, W.A. The genetics of the Rhesus blood group system. *Blood Transfus* 2007;5(2):50-57.

Garte, S.*, et al.* Metabolic gene polymorphism frequencies in control populations. *Cancer Epidemiol Biomarkers Prev* 2001;10(12):1239-1248.

Grubisa, I.*, et al.* Combined GSTM1 and GSTT1 null genotypes are strong risk factors for atherogenesis in a Serbian population. *Genet Mol Biol* 2018;41(1):35-40.

Kasthurinaidu, S.P.*, et al.* GST M1-T1 null allele frequency patterns in geographically assorted human populations: a phylogenetic approach. *PLoS One* 2015;10(4):e0118660.

Nath, S.*, et al.* The GSTM1 and GSTT1 null genotypes increase the risk for Type 2 diabetes mellitus and the subsequent development of diabetic complications: A meta-analysis. *Curr Diabetes Rev* 2017.

Petrovic, D. and Peterlin, B. GSTM1-null and GSTT1-null genotypes are associated with essential arterial hypertension in patients with type 2 diabetes. *Clin Biochem* 2014;47(7-8):574-577.

Wang, M.*, et al.* GSTM1 Null Genotype and GSTP1 Ile105Val Polymorphism Are Associated with Alzheimer's Disease: a Meta-Analysis. *Mol Neurobiol* 2016;53(2):1355-1364.
